# Intraluminal dural venous sinus cyst simulating as aerocele in computerized tomography brain

**DOI:** 10.4103/0972-2327.41881

**Published:** 2008

**Authors:** Rajul Rastogi

**Affiliations:** Yash Diagnostic Center, Yash Hospital and Research Center, Civil Lines, Kanth Road, Moradabad, UP - 244 001, India

**Keywords:** Aerocele, cyst, venous sinus

## Abstract

Intradural venous sinuses are commonly visualized structures in the CT brain, even in noncontrast images. Rarely, hypoattenuating focal lesions may be observed within their lumen as a coincidental finding, which may remain undiagnosed. However, when such lesions appear in the patients who are scanned for head injury, they might pose diagnostic difficulties

## Introduction

Intradural venous sinus cysts are very uncommon. They are mainly congenital. They may be simple cysts or dermoid cysts. Usually, they are asymptomatic and of no consequence, hence often overlooked. However, they may be symptomatic as well. They might pose difficulty in patients with head injury when they may fallaciously be diagnosed as aeroceles in noncontrast CT brain. This might lead to unnecessary search for sinus or compound fractures, which ultimately proves to be futile.

Through this brief case report, the author aims to enrich the knowledge about these rare lesions, their important differential diagnosis and their importance.

## Case Report

A 30-year-old male underwent CT head to rule head injury.

Noncontrast study of the brain was performed by transaxial scanning from the base of the skull to the vertex in the sequential mode. Brain parenchyma was apparently normal. However, two subcentimeter hypoattenuating focal lesions that were well-defined and oval to rounded were noted in the straight sinus and the torcular herophili region of the dural venous sinuses. As the patient was a case of head injury, the diagnosis of aeroceles was considered. However, even close examination at the bone window images failed to reveal any evidence of sinus fracture or compound fracture of the skull. The patient was then taken for the CT scan of the paranasal sinus region with thinner slices; however, they also failed to reveal any breach in the cortex of any of the paranasal sinus. Closer review of the images of the head region revealed that the lesions were having a fluid attenuation (20–30 HU).

**Figure 1:**
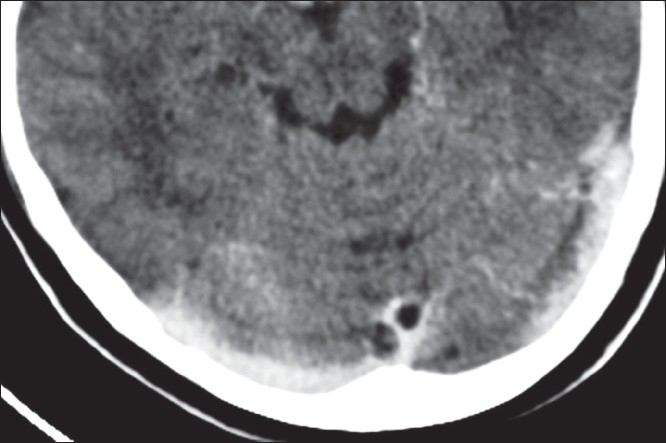
Transaxial CECT image showing the dural venous sinus cyst in the straight sinus

**Figure 2:**
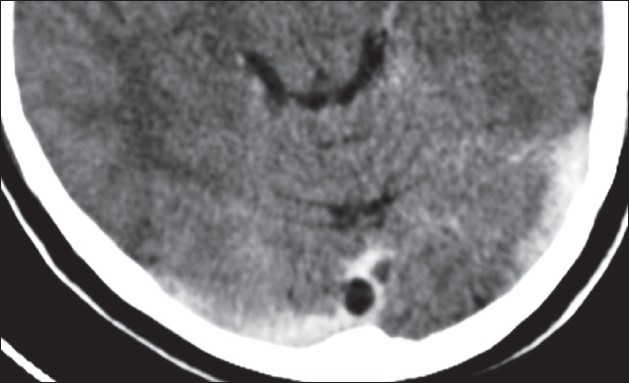
Transaxial CECT image showing the dural venous sinus cyst in the torcular herophili

**Figure 3:**
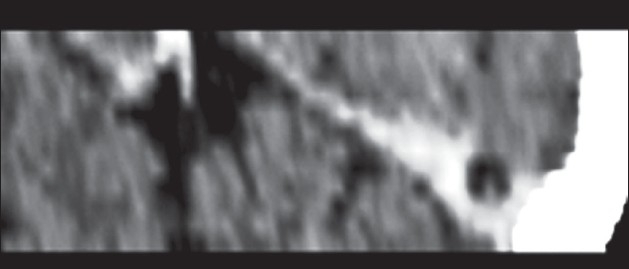
Sagittal MPR CECT image showing the dural venous sinus cyst in the torcular herophili

**Figure 4:**
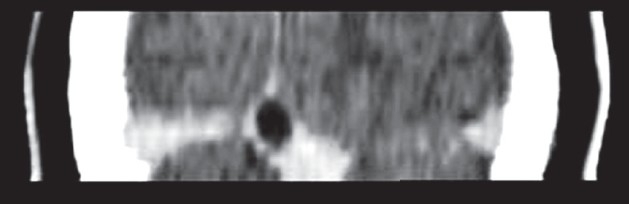
Coronal MPR CECT image showing the dural venous sinus cyst in the torcular herophili

For better delineation of the lesions, the patient underwent contrast enhanced CT brain, which was performed in the spiral mode with multiplanar reconstruction in different planes. This study delineated the lesions better and due to adequate contrast, they were finally diagnosed asintradural simple venous sinus cysts [Figures 1–4]. There was no evidence of dilatation of the venous sinuses. There was no postcontrast enhancement.

## Discussion

Intraluminal dural venous sinus cysts are very rare congenital malformations.[1] They are usually simple cysts but may occasionally be dermoid cysts when they represent the extension of the dorsal dermal sinus syndrome. They are usually asymptomatic and incidental. However, when large, they may produce symptoms secondary to obstruction to the venous outflow. The usual symptoms are headache but may also cause syncope.[2] The occlusion of venous drainage can cause rapid elevation in the intracranial pressure. In a younger age group, such obstructing lesions may lead to enlargement of the head and developmental retardation.[1]

Similar to any other cystic lesion in the body, these lesions also appear fluid attenuating, i.e., 0–30 HU and isodense to cerebrospinal fluid in the CT scan of the brain. In postcontrast images, they are observed as intraluminal filling defects with no uptake of contrast or change in attenuation values over the noncontrast images. On MRI, these lesions appear isointense to the cerebrospinal fluid in all sequences, i.e., hypointense in T1-weighted and FLAIR images and hyperintense in T2-weighted images. Postgadolinium images reveal their intraluminal location with no evidence of contrast enhancement.[2] CT or MR venography is better in delineating their intraluminal location and extent where they areidentified as filling defects within the dural venous sinus. There is usually no evidence of proximal venous sinus dilatation unless the lesions are sufficiently big to cause substantial obstruction to the venous outflow.

Although majority of these lesions are incidentally detected and are usually asymptomatic, they may yet require conservative or surgical management in symptomatic patients. Medical therapy may be sufficient to control headaches in majority of the cases. However, frequent episodes of syncope is usually an indication of surgical excision of these lesions.[2]

The important differential diagnosis involves adipose tissue, arachnoid granulations and aeroceles.

The presence of adipose tissue has been reported in the dural sinuses by many authors.[3,4] Their fat density easily differentiates them in both the CT and MR images of brain.

Arachnoid granulations can be visualized within the sinuses, including the posterior sagittal sinus.[5,6] Occasionally, these can become quite large, even mimicking tumors. These granulations appear as sessile or pedunculated polypoidal structures attached to the walls of the sinus in the specific locations.

Although aeroceles may visually be mistaken for cysts, their close examination reveal CT densities of less than -50 HU and they appear as signal voids in all sequences of MR imaging.

Very few references are present in the medical literature regarding these peculiar but simple to diagnose lesions, particularly the symptomatic ones.[2]

To summarize, Intraluminal dural cysts are rare lesions that are usually detected incidentally, but they should be differentiated from other similar appearing lesion and should be reported as they may be symptomatic in the future.
